# Exploring the association between grade point average and work engagement: insights from a cross-sectional study of medical students

**DOI:** 10.3389/fmed.2025.1536482

**Published:** 2025-10-08

**Authors:** Runzhi Huang, Jiajie Zhou, Yang Chen, Wei Zhang, Min Lin, Meiqiong Gong, Bingnan Lu, Minghao Jin, Yuntao Yao, Yuwei Lu, Xirui Tong, Jianyu Lu, Maosheng Yu, Huabin Yin, Xiaonan Wang, Xin Liu, Yue Wang, Wenfang Chen, Chongyou Zhang, Erbin Du, Qing Lin, Zongqiang Huang, Jie Zhang, Yifan Liu, Dayuan Xu, Shuyuan Xian, Shizhao Ji

**Affiliations:** ^1^Department of Burn Surgery, The First Affiliated Hospital of Naval Medical University, Shanghai, China; ^2^Research Unit of Key Techniques for Treatment of Burns and Combined Burns and Trauma Injury, Chinese Academy of Medical Sciences, Shanghai, China; ^3^Shanghai Jiao Tong University School of Medicine, Shanghai, China; ^4^Department of Anesthesiology, Shanghai Pulmonary Hospital Affiliated to Tongji University, Shanghai, China; ^5^Mental Health Education and Consultation Center, Chongqing Medical University, Chongqing, China; ^6^Office of Educational Administration, Shanghai University, Shanghai, China; ^7^Department of Urology, Xinhua Hospital Affiliated to Shanghai Jiao Tong University School of Medicine, Shanghai, China; ^8^Department of Orthopedics, Shanghai General Hospital, School of Medicine, Shanghai Jiaotong University, Shanghai, China; ^9^Department of Epidemiology and Health Statistics, School of Public Health, Capital Medical University, Beijing, China; ^10^Department of Rheumatology and Immunology, Second Affiliated Hospital of Naval Medical University, Shanghai, China; ^11^Department of Health Statistics, School of Public Health, Air Force Medical University, Xi'an, China; ^12^Faculty of Medicine, Jinggangshan University, Ji’An, China; ^13^Basic Medical College, Harbin Medical University, Harbin, Heilongjiang, China; ^14^Frist Clinical Medical College, Mudanjiang Medical University, Mudanjiang, China; ^15^Department of Human Anatomy, Laboratory of Clinical Applied Anatomy, School of Basic Medical Sciences, Fujian Medical University, Fuzhou, China; ^16^Department of Orthopedics, The First Affiliated Hospital of Zhengzhou University, Zhengzhou, China; ^17^Department of Gynecology, Shanghai First Maternity and Infant Hospital, Tongji University School of Medicine, Shanghai, China

**Keywords:** medical students, work engagement (WE), grade point average (GPA), Utrecht Work Engagement Scale (UWES), cross-sectional study

## Abstract

**Background:**

Medical students’ work engagement (MSWE) is widely considered an essential indicator of their state of mind, affecting their productivity and future career development as doctors. In our previous research, grade point average (GPA) was demonstrated to be an independent predictor of self-regulated learning (SRL), which is closely associated with MSWE. However, the relationship between GPA and MSWE has not been systematically elucidated. Our study aims to discover and clarify the significant association between GPA and MSWE.

**Methods:**

We collected data from 12 universities in China and evaluated MSWE using the Utrecht Work Engagement Scale (UWES). Then, we conducted a cross-sectional study where GPA and UWES scores or categories were recorded simultaneously. Pearson’s chi-squared tests and Welch’s ANOVA were utilized to explore the distributional association between GPA and MSWE. Multivariate logistic regression analysis was performed to examine whether GPA was a significant factor of MSWE, followed by a subgroup analysis to exclude other confounding factors. Ultimately, GPA was used as a key variable to develop a nomogram aimed at evaluating the possibility of low UWES scores, along with calibration and accuracy assessments.

**Results:**

Pearson’s chi-squared tests (*p* = 2.54e-65) and Welch’s ANOVA (*p* = 8.07e-48) demonstrated a strong association between GPA and UWES scores, indicating a significant relationship with MSWE. Medical students with a GPA in the “top 5%” and “5–20%” categories exhibited a higher level of MSWE. Multivariate analysis revealed that GPA was statistically significant across all rank categories (all *p* < 0.001), thereby + GPA’s significance in factoring MSWE. In addition, statistical significance persisted in subgroup analysis, which excluded the confounding effect of age and gender. Ultimately, the nomogram was validated as accurate and reliable (area under the curve (AUC) = 0.626), providing a quantitative assessment of MSWE primarily based on GPA.

**Conclusion:**

Medical students with higher GPA scores tended to exhibit better MSWE. GPA was strongly validated as a significant factor in evaluating MSWE.

## 1 Introduction

Work engagement (WE) is conceptualized as a positive, fulfilling, and affective-motivational state of mind experienced during work (1). WE refers to a more persistent and pervasive state that is not focused on any particular object, event, individual, or behavior but on an overall work environment and an everlasting work stage (2, 3). WE can be conceptualized through three dimensions: vigor (high levels of energy and mental resilience), dedication (being strongly involved in one’s work), and absorption (being fully concentrated and happily engrossed in one’s work) (4). Furthermore, WE is widely considered to have a strong association with various work-related outcomes and qualities that reflect one’s work performance and productivity (5, 6), thereby serving as a good indicator for predicting one’s capability and contribution to the team (7). A person with a higher level of WE is expected to have more enthusiasm and a creative commitment to work, while a low level of WE suggests a higher risk of burnout and inefficacy.

Medical students’ work engagement (MSWE) turns the focus particularly on the group of medical students, emphasizing their theoretical learning of medicine and practical training of relevant skills. Previous research states that MSWE could serve as a positive construct for exploring medical students’ well-being (8). It is noteworthy that the discipline of medicine is regarded as one of the toughest fields to study, which can be attributed to several factors. First, medical students are expected to update their medical knowledge and keep pace with every breakthrough in medical techniques (9, 10). Second, medical students should know how to manage their relationships with patients, which tests their professionalism, empathy, and communication skills (11). Third, medicine is a field concerning application with the goal of pain relief, health promotion, and disease prevention (12), directly impacting citizens’ basic right to life and health. Most importantly, MSWE plays an important role in medical education. Based on the particularity of medicine, engaged medical students are more likely to not only actively participate in clinical procedures and internalize the professional values but also create a collaborative learning culture and cope with challenges (13, 14). As the healthcare landscape continues to evolve, medical students who are engaged in their education are better prepared to adapt to changes such as centered care, innovative research, and multidimensional development. Conclusively, MSWE affects clinical skill development, professional identity formation, the learning environment, burnout prevention, and future contributions to healthcare. Therefore, MSWE is overwhelmingly essential for the professional, moral, and behavioral education of medical students. Furthermore, it is also significant to identify the decisive factors influencing MSWE and to develop effective strategies to promote MSWE.

In our previous studies (15–20), we developed a comprehensive nomogram model to predict self-regulated learning (SRL) levels among Chinese medical undergraduates. Specifically, “GPA,” the abbreviation of grade point average, was demonstrated to be an independent predictor of SRL levels. Fundamental evidence has been established to show the close relationship between SRL levels and WE (21, 22). However, the relationship between GPA and MSWE has not yet been clearly elucidated (15). GPA is imperatively recognized as an overall measure of students’ performance and capability in terms of synthetic learning. Therefore, our study aimed to identify and clarify the significant association between GPA and MSWE. Meanwhile, we further analyzed the potential reasons behind our findings and proposed some practical strategies to ameliorate MSWE.

Our approach to evaluating MSWE was to utilize the Utrecht Work Engagement Scale (UWES), which contains three dimensions and 16 items. The UWES was selected as a gold-standard tool with validated psychometric properties for measuring work engagement across cultures (23). Its 16-item structure captures three theoretically grounded dimensions—vigor, dedication, and absorption—that align with our study’s focus on medical students’ multidimensional engagement. The scale has demonstrated excellent reliability (Cronbach’s *α* > 0.90) and construct validity in previous studies on medical education (8, 24). The UWES has been widely adopted in over 30 studies investigating healthcare trainees’ engagement (25, 26), making our results comparable to existing literature. The final scores were calculated by summing the responses to each item. Then, we divided medical students into high and low UWES cohorts based on their median UWES scores. Next, we conducted a cross-sectional study where GPA and UWES scores or categories were recorded at the same time. Afterward, we performed Pearson’s chi-squared test and Welch’s ANOVA to confirm that a medical student with a higher GPA is strongly associated with WE in learning. We aimed to figure out the factors influencing MSWE and provide evidence for further exploration, thereby promoting the improvement of medical education.

## 2 Materials and methods

### 2.1 Sample source and data extraction

This research received approval from the Ethics Committee of the First Affiliated Hospital of Naval Medical University.

A cross-sectional study was conducted among medical students from 12 representative universities in mainland China. These universities were divided into four categories: 985 Project Universities (Peking University and Tongji University), 211 Project Universities (Zhengzhou University), Military Universities (Air Force Medical University and Naval Medical University), and Non-985/211 Project Universities (Jinggangshan University), including the first batch of medical universities (Capital Medical University, Fujian Medical University, Southwest Medical University, Chongqing Medical University, and Harbin Medical University) and the second batch of medical universities (Mudanjiang Medical College).

Prior to distributing the formal questionnaire, a pilot study was conducted with 20 voluntary undergraduate students who were randomly selected to fill out the questionnaire and provide some constructive feedback. Then, we revised the questionnaire in accordance with each student’s feedback to ensure the quality and clarity of the questions listed. Afterward, the questionnaire was uploaded to Wenjuanxing,^1^ an online survey platform. The electronic questionnaire was converted into a link and sent to the heads of 12 target medical schools. Ultimately, a stratified cluster random sampling method was implemented, using grade level as the stratifying factor. In each grade at each university (from Grade 1 to Grade 5, with the remaining students grouped as graduates), 150 students were selected by lottery to fill out the questionnaire, which was released in the same format. Data with outliers or missing values were excluded from the analysis. Apart from basic demographic information (age, gender, and native place), GPA, our interested variable, was recorded based on the medical students’ ranking within their major (Top 5%, 5–20%, 20–50%, 50–80%, and 80–100%). In addition, other minor factors, such as the doctor–patient relationship, were also labeled as characteristics, which were further listed in the format of a heatmap.

### 2.2 Instrument to evaluate medical students’ work engagement

In this research, the UWES was applied to evaluate medical students’ study engagement. In previous longitudinal studies, researchers from the Netherlands have verified the validity of the UWES score in predicting long-term mental health (25), which aligns with the content of MSWE. Moreover, a previous study analyzed the validity evidence and reliability of the UWES in medical students (27), further supporting the scale’s favorable internal reliability and structural validity. The UWES contains three dimensions (vigor, dedication, and absorption) and 16 items of subjective statements (26), which require target students to make self-assessments based on their own situation. Each item is assessed using a seven-point Likert scale ranging from never (0 point) to always (6 points), which corresponds to the dimension of the UWES. Therefore, the UWES scores not only assess an individual’s mental status in terms of study engagement but also reflect the degree of each dimension. In general, a higher score means a more positive and fulfilling mental state of study engagement.

### 2.3 Pearson’s chi-squared test and Welch’s ANOVA

Initially, for descriptive statistical analysis, continuous variables were expressed as means (with standard deviations, SD) or medians (with interquartile ranges, IR), and categorical variables were presented as counts (with percentages). To quantify MSWE, the UWES scores were categorized using the median cutoff value of 72 points, with scores below 72 classified as low and scores of 72 or higher classified as high. Pearson’s chi-squared test was conducted to explore the statistical significance of the UWES categories within each GPA group and overall distribution. The results of the Pearson’s chi-squared test were presented in the following boxplot, with the chi-squared value and *p*-value listed. Meanwhile, Welch’s ANOVA was conducted to figure out the correlation between GPA and UWES scores, as well as the statistical significance of UWES scores between GPA groups. The results of the Welch’s ANOVA were visualized using a violin plot, where the mean score for each GPA group and *p*-values were also labeled. To avoid the impact of confounding factors, age and gender were used as subgroups after the primary classification of GPA groups. Therefore, Pearson’s chi-squared test and Welch’s ANOVA were re-performed to estimate the statistical significance of the UWES scores across age and gender subgroups. The results were also visualized using boxplots and violin plots.

### 2.4 Multivariate logistic regression analysis

In the univariate analysis, the correlation between GPA and the UWES was preliminarily explored. Considering the comprehensive influence of multiple variables on the UWES, we also performed a multivariate logistic regression analysis to estimate whether GPA is a significant factor in the UWES. Variables including age, gender, ethnicity, major, grade, native place, and, most importantly, GPA were taken into consideration. For each variable, the odds ratio (OR) was calculated to evaluate the influence on the UWES. Therefore, sets of ORs, coupled with a corresponding 95% confidence interval (95% CI) and *p*-values, are listed in a table.

### 2.5 Development and validation of a nomogram

In our study, a nomogram was established to evaluate the probability of low UWES scores, where the seven variables mentioned in the multivariate analysis were included. In the nomogram, the scores of each variable were visualized into lines. To validate the accuracy of the model presented by the nomogram, receiver operating characteristic (ROC) curves and calibration curves were used to evaluate its discrimination and calibration performance. ROC analysis evaluates discriminative ability using all possible thresholds, with Youden’s index used to determine the optimal cutoff. In the calibration curve, the Hosmer–Lemeshow test was performed to quantify the model’s goodness of fit, while the slope of the calibration curve was used to indicate whether the evaluative model was overfitting. Meanwhile, by drawing the decision curve analysis (DCA) and comparing it with the ideal curve, the net benefit was calculated to quantify the clinical utility of the nomogram under different decision thresholds.

### 2.6 Statistical analysis

All statistical analyses and corresponding curves were performed using R version 4.2.2 (Institute for Statistics and Mathematics, Vienna, Austria) and SPSS 20.0 (SPSS Inc., Chicago, IL, USA). A two-sided *p*-value of < 0.05 was considered statistically significant.

## 3 Results

### 3.1 Sample characteristics

**Figure 1:**
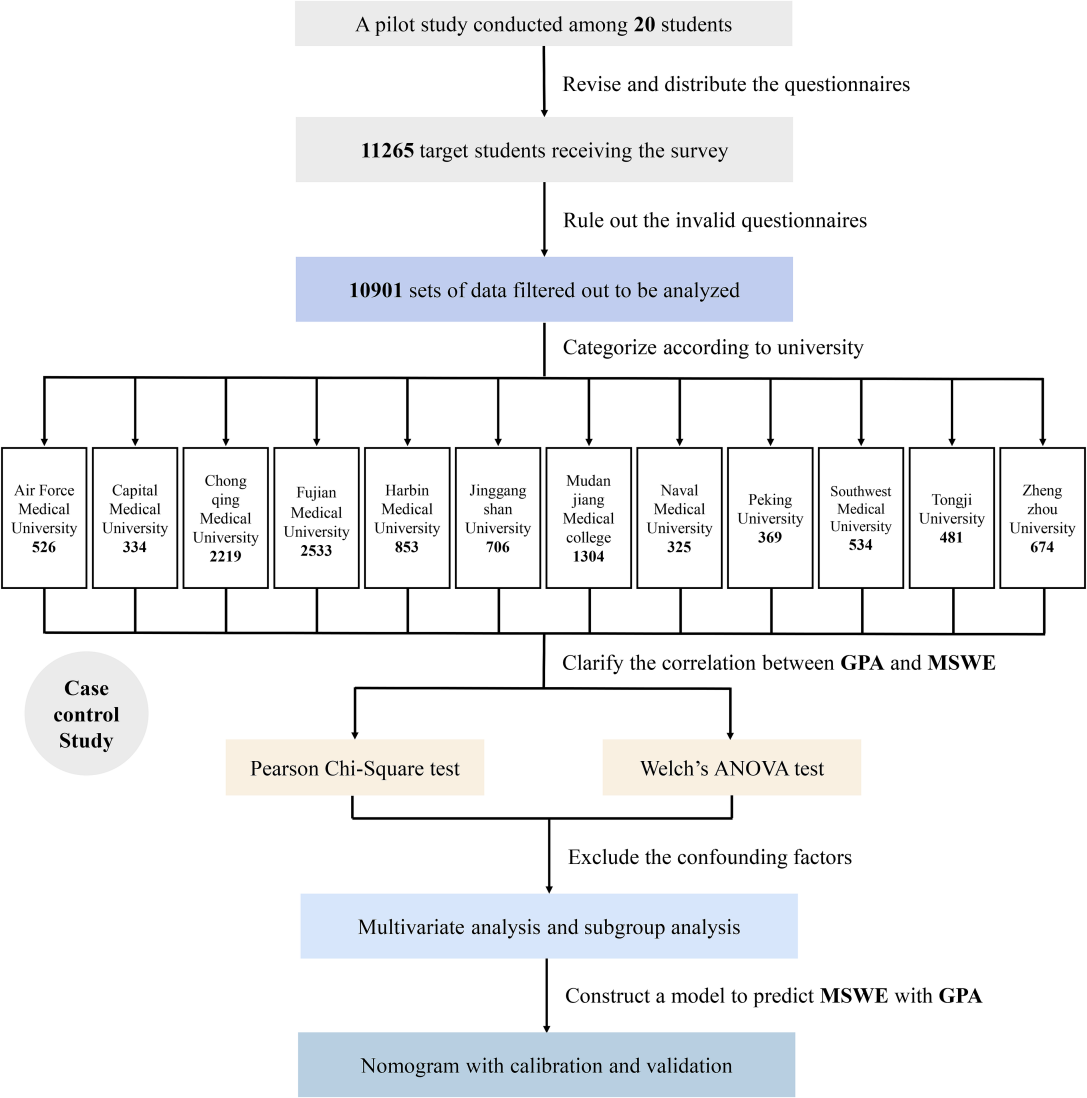
Flowchart describing how the research was designed and conducted, ranging from sample data extraction and statistical analysis to result validation and model construction.

**Figure 2:**
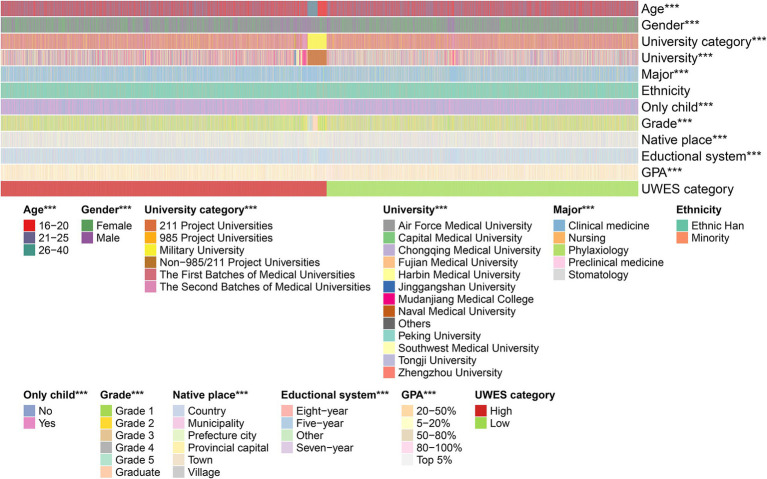
Heatmap visualizing the distribution of the target students’ answers to each single question in the questionnaire. The students’ UWES scores were divided into low and high categories. Statistical significance for each variable across the UWES categories is indicated by star icons, and three stars represent a *p*-value <0.001.

A total of 11,265 target students submitted the questionnaire and completed the UWES (Supplementary Table S1), but only 10,901 responses were filtered and included in the further analysis (Figure 1). Their general information is listed in a Table 1 and visualized as heatmaps (Figure 2), along with their answers.

**Table 1:** Characteristics of 10,901 participants.

Variables number (percent)	
Age	
16–20	5,868 (53.83)
21–25	4,825 (44.26)
26–40	208 (1.91)
Gender	
Female	6,531 (59.91)
Male	4,370 (40.09)
University category	
211 Project Universities	692 (6.35)
985 Project Universities	853 (7.82)
Military University	851 (7.81)
Non-985/211 Project Universities	720 (6.6)
The First Batches of Medical Universities	6,473 (59.38)
The Second Batches of Medical Universities	1,312 (12.04)
University	
Air Force Medical University	526 (4.83)
Capital Medical University	334 (3.06)
Chongqing Medical University	2,219 (20.36)
Fujian Medical University	2,533 (23.24)
Harbin Medical University	853 (7.82)
Jinggangshan University	706 (6.48)
Mudanjiang Medical College	1,304 (11.96)
Naval Medical University	325 (2.98)
Peking University	369 (3.39)
Southwest Medical University	534 (4.9)
Tongji University	481 (4.41)
Zhengzhou University	674 (6.18)
Others	43 (0.39)
Major	
Clinical medicine	8,668 (79.52)
Nursing	572 (5.25)
Phylaxiology	698 (6.4)
Preclinical medicine	658 (6.04)
Stomatology	305 (2.8)
Ethnicity	
Ethnic Han	10,190 (93.48)
Minority	711 (6.52)
Only child	
No	6,140 (56.33)
Yes	4,761 (43.67)
Grade	
Grade 1	3,800 (34.86)
Grade 2	2043 (18.74)
Grade 3	1,666 (15.28)
Grade 4	1869 (17.15)
Grade 5	1,298 (11.91)
Graduate	225 (2.06)
Native place	
Country	2,562 (23.5)
Municipality	1,535 (14.08)
Prefecture	2063 (18.92)
Provincial capital	1,127 (10.34)
Town	1,196 (10.97)
Village	2,418 (22.18)
Educational system	
Eight-year	1,305 (11.97)
Five-year	7,621 (69.91)
Seven-year	280 (2.57)
Other	1,695 (15.55)
GPA	
Top 5%	815 (7.48)
5–20%	2,509 (23.02)
20–50%	3,844 (35.26)
50–80%	2,687 (24.65)
80–100%	1,046 (9.6)
Father’s educational level	
Preliminary school	1749 (46.46)
Junior high school	3,800 (34.86)
Senior high school	2,623 (24.06)
Junior college	1,141 (10.47)
Bachelor degree	1,292 (11.85)
Graduate degree	251 (2.3)
Father’s occupation	
Civil servant	1,083 (9.93)
Company employee	1,093 (10.03)
Freelance work	2,112 (19.37)
Individual household	1,092 (10.02)
Professional/technical	1,150 (10.55)
Worker/peasant	4,371 (40.1)
Mother’s educational level	
Preliminary school	3,180 (29.17)
Junior high school	3,322 (30.47)
Senior high school	2,249 (20.63)
Junior college	1,017 (9.33)
Bachelor degree	959 (8.8)
Graduate degree	174 (1.6)
Mother’s occupation	
Civil servant	634 (5.82)
Company employee	1,250 (11.47)
Freelance work	2,982 (26.53)
Individual household	791 (7.26)
Professional/technical	1,363 (12.5)
Worker/peasant	3,971 (36.43)
Learning environment of your schools	
Terrible	63 (0.58)
Bad	125 (1.15)
Common	2,284 (20.95)
Good	6,048 (55.48)
Excellent	2,284 (20.95)
Doctor-patients relationship in your hospitals	
Terrible	46 (0.42)
Bad	121 (1.11)
Common	2,654 (24.35)
Good	6,190 (56.78)
Excellent	1732 (15.89)
Interests of medicine	
Extremely uninterested	65 (0.6)
Uninterested	165 (1.51)
Common	2,654 (24.35)
Interested	6,145 (56.37)
Extremely interested	1872 (17.17)
UWES category	
High scores (≥72)	5,572 (51.11)
Low scores (<72)	5,329 (48.89)

GPA, grade point average; UWES, Utrecht Work Engagement Scale.

According to the sample data, the majority of the medical students were between 16 and 25 years (98.09%). There were more female students (19.82%) than male students. More than half of the target students were from the first batch of medical universities and clinical medicine was the most common major. Grade 1 students (34.86%) formed the primary cluster in our research. Most importantly, 7.48% of the medical students ranked in the top 5% of GPA, while those with a GPA ranking between the top 5 and 20% accounted for 23.02%. Meanwhile, 35.26% of the samples were within the GPA ranking range of 20–50%. In addition, 24.65 and 9.6% of the students were in the 50–80% and 80–100% GPA ranking blocks, respectively. In addition, the students’ parents had a low level of education in general. In most students’ opinion, they enjoyed a good learning environment at their schools. Ultimately, the UWES scores were non-normally distributed and did not meet the homogeneity of variance according to the F-test. Therefore, the UWES scores were empirically divided at the median cutoff value into a low category (score < median, 48.89%) and a high category (score ≥ median, 51.11%).

### 3.2 Significant correlation between medical students’ GPA and UWES categories and scores

**Figure 3:**
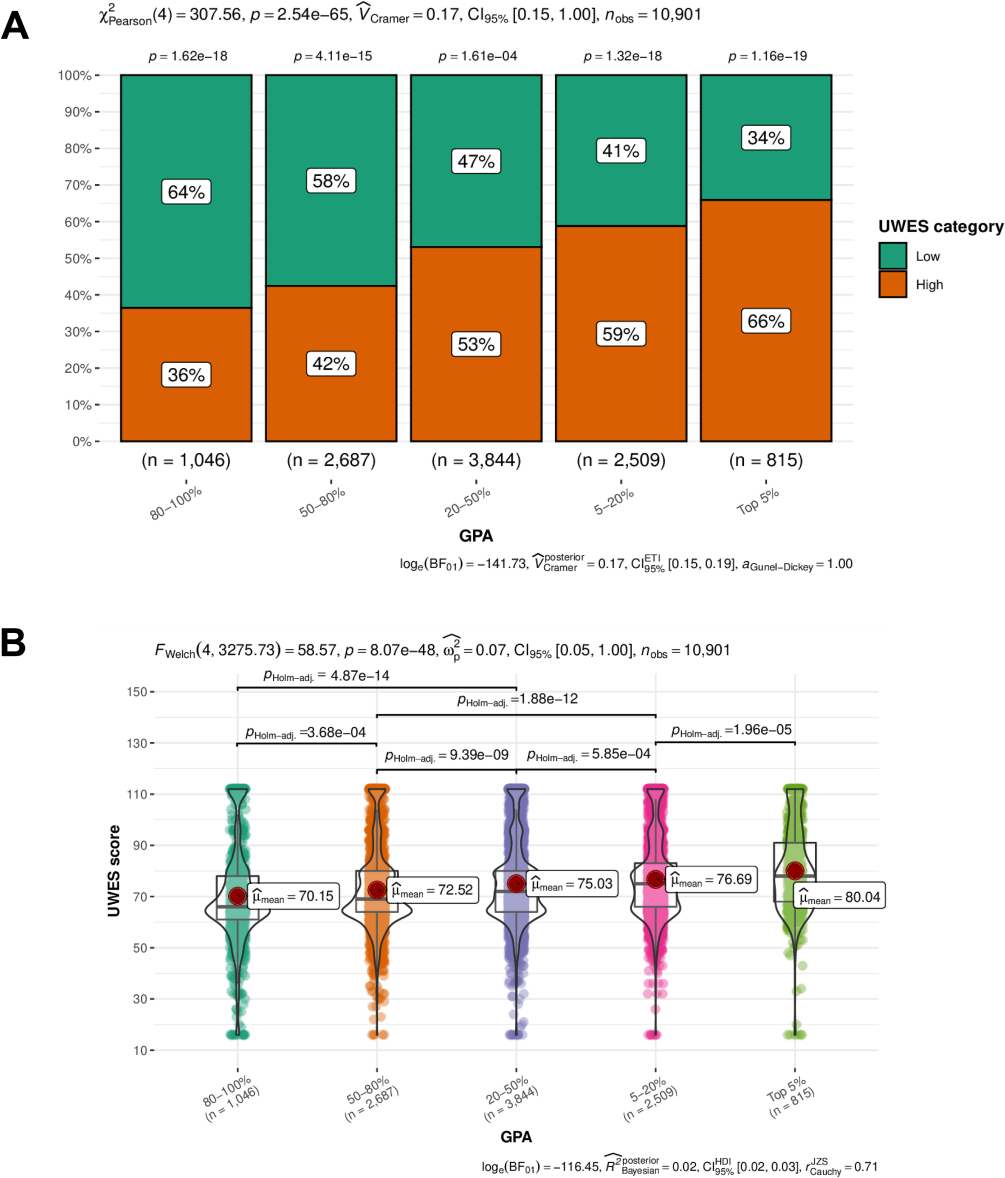
Results of the univariate analysis illustrating the relationship between GPA (exposure) and UWES scores and categories. **(A)** The results of Pearson’s chi-squared test visualized in a boxplot. Vertical and horizontal axes stand for the percentage of the UWES score and GPA category, respectively. The UWES categories exhibited a statistically significant distribution within each GPA group (*p* < 0.05). **(B)** The results of Welch’s ANOVA visualized in a violin plot. Vertical and horizontal axes stand for the UWES score and GPA category, respectively. The medical students with higher GPAs tended to have a higher median UWES score. UWES, Utrecht Work Engagement Scale; GPA, grade point average.

Similarly, due to the non-normal distribution and heterogeneity of variance, we used non-parametric tests for further analysis. To clarify the correlation between the medical students’ GPA and the UWES categories and scores, Pearson’s chi-squared test and Welch’s ANOVA were used to verify the significant correlation directly. Figure 3A demonstrates that the UWES categories exhibited statistically significant distribution within each GPA group (*p* < 0.05). A preliminary conclusion can be drawn that there is a significant correlation between medical students’ GPA and the UWES categories. In detail, the percentage of students in the low UWES category significantly outweighed that of the high UWES category in the GPA groups of “80–100%” and “50–80%.” In contrast, the high UWES category predominated in the remaining GPA groups. It was revealed that the medical students with better GPAs had higher UWES scores. In addition, the significance of the UWES category composition turned out to be more apparent at both the high and low extremes of GPA rankings.

In Figure 3B, the violin plots and scatter plots demonstrate statistical significance, showing mean scores and *p*-values between each GPA group. From the perspective of mean scores, a general trend could be observed: the better the GPA, the higher the UWES scores. To further explore whether such a trend was statistically significant, Welch’s ANOVA was conducted across the different GPA groups, from which we could tell that the significance existed in each intergroup combination. Consequently, the conclusion was further supported: the medical students with higher GPAs tended to have higher UWES scores.

Overall, GPA was proved to be an important factor positively correlated with MSWE. The students in the top 5% GPA group showed a high likelihood of positive MSWE, while those in the 80–100% GPA range were more likely to be associated with negative MSWE. This is likely because medical students with high GPAs always tend to be highly motivated to achieve, possess extensive medical knowledge and clinical skills, and demonstrate better self-regulation, outstanding interpersonal skills, and a spirit of collaboration. Further interpretation is provided in the discussion section.

### 3.3 Significant correlation between GPA and MSWE

Having identified a correlation between GPA and MSWE, we performed multivariate logistic regression analysis and subgroup analysis to exclude the interference from confounding factors, thus assessing whether the correlation between GPA and MSWE was significant. Table 2 shows the OR (95% CI) and *p*-values for each variable category. Although the stomatology major turned out to be a significantly protective factor (OR = 0.764 < 1) compared to clinical medicine, the evidence was still insufficient to verify a correlation between major and the UWES scores because of the lack of significance observed in preclinical medicine (*p* = 1.033 > 0.05). Similarly, we were also able to exclude confounding effects from ethnicity, grade level, and native place. It is noteworthy that GPA demonstrated a strong significant association with the UWES scores, as the p-values for each GPA rank category were all below 0.001. Taking “GPA 20-50%” as reference, groups of “GPA Top 5%” (OR = 0.567 <1) and “GPA 5-20%” (OR = 0.766<1) were demonstrated to protect medical students out of the low UWES, while groups of “GPA 50-80%” (OR = 1.559) and “GPA 80–100%”(OR = 2.064) showed the tendency of low UWES.

**Table 2:** Multivariate logistic regression analysis of UWES scores.

Variable	UWES scores	
	OR (95% CI)	*p*-value
Age		
16–20	1.00 (reference)	
21–25	0.866 (0.754–0.995)	0.042^*^
26–40	0.572 (0.396–0.827)	0.003^*^
Gender		
Female	1.00 (reference)	
Male	0.705 (0.650–0.765)	<0.001^*^
Ethnicity		
Ethnic Han	1.00 (reference)	
Minority	0.906 (0.775–1.061)	0.220
Major		
Clinical medicine	1.00 (reference)	
Nursing	1.461 (1.218–1.752)	<0.001^*^
Phylaxiology	1.293 (1.102–1.517)	0.002^*^
Preclinical medicine	1.033 (0.878–1.216)	0.697
Stomatology	0.764 (0.601–0.969)	0.026^*^
Grade		
Grade 1	1.00 (reference)	
Grade 2	1.287 (1.150–1.441)	<0.001^*^
Grade 3	1.499 (1.289–1.742)	<0.001^*^
Grade 4	1.597 (1.341–1.901)	<0.001^*^
Grade 5	1.142 (0.945–1.379)	0.169
Graduate	1.463 (0.032–0.729)	0.032^*^
Native place		
County	1.00 (reference)	
Municipality	1.022 (0.805–1.078)	0.743
Prefecture city	0.780 (0.692–0.879)	<0.001^*^
Provincial capital	0.767 (0.664–0.886)	<0.001^*^
Town	0.971 (0.843–1.117)	0.679
Village	0.943 (0.841–1.057)	0.313
GPA		
GPA 20–50%	1.00 (reference)	
GPA 5–20%	0.766 (0.691–0.850)	<0.001^*^
GPA 50–80%	1.559 (1.410–1.725)	<0.001^*^
GPA 80–100%	2.064 (1.788–2.383)	<0.001^*^
GPA Top 5%	0.567 (0.483–0.665)	<0.001^*^

UWES, Utrecht Work Engagement Scale; GPA, grade point average; OR, odds ratio; CI, confidence interval. ^*^p < 0.05.

**Figure 4:**
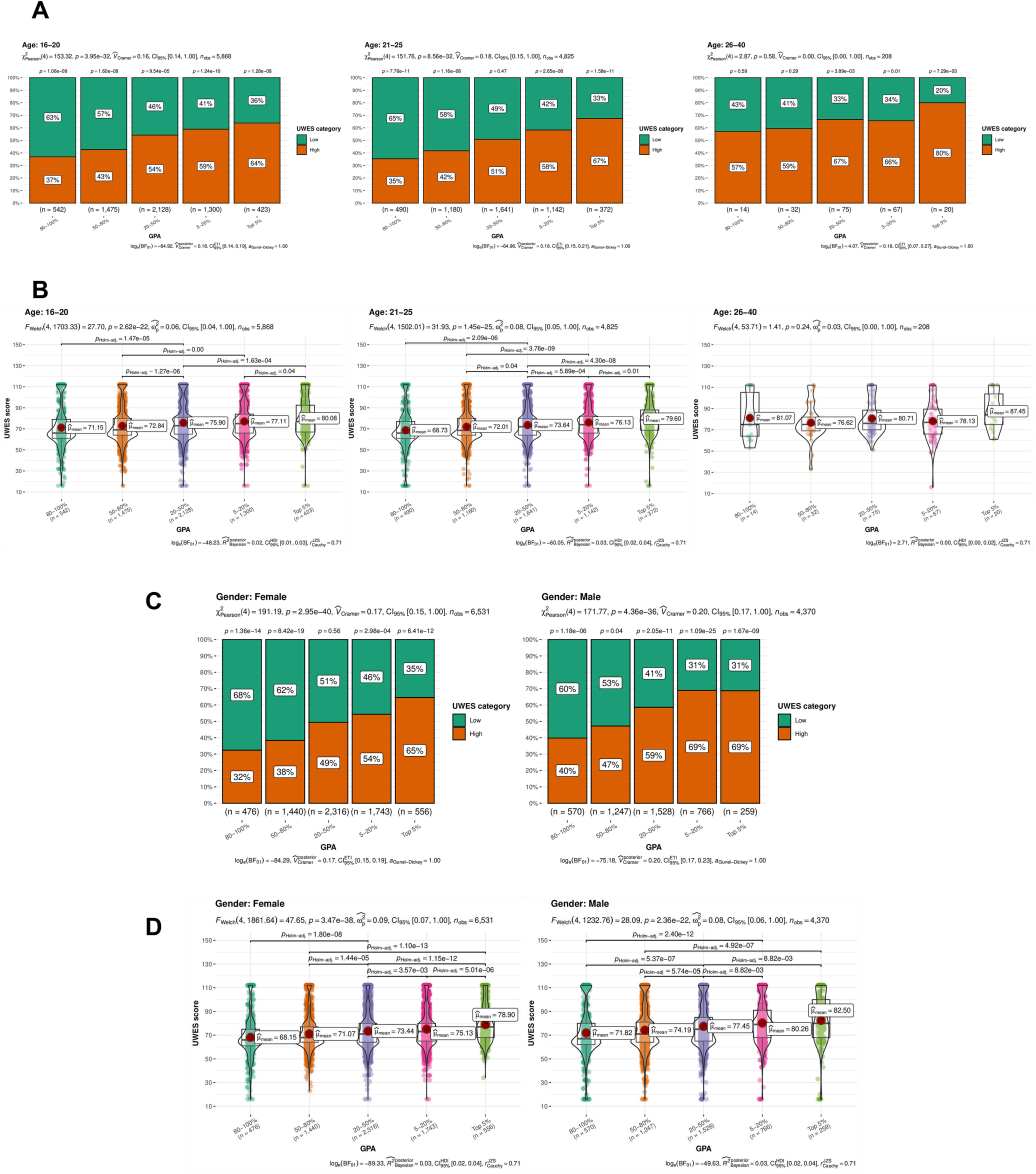
Results of the subgroup analysis, excluding the confounding effects of age and gender. **(A)** The results of Pearson’s chi-squared test for age subgroups, visualized in boxplots. Vertical and horizontal axes stand for the percentage of the UWES score and GPA category, respectively. The group was age category. Statistical significance of the UWES categories was still present within the age subgroups of 16–20 (*p* = 3.95e-32) and 21–25 (*p* = 8.56e-32). **(B)** The results of Welch’s ANOVA for age subgroups, visualized in violin plots. Vertical and horizontal axes stand for the UWES score and GPA category, respectively. The group was age category. Statistical significance of the UWES scores was still present within the age subgroups of 16–20 (*p* = 2.62e-22) and 21–25 (*p* = 1.45e-25). **(C)** The results of Pearson’s chi-squared test for gender subgroups, visualized in boxplots. Vertical and horizontal axes stand for the percentage of the UWES score and GPA category, respectively. The group was gender. The UWES categories still showed statistical significance in the women (*p* = 2.95e-40) and men (*p* = 4.36e-36) subgroups. **(D)** The results of Welch’s ANOVA for gender subgroups, visualized in violin plots. Vertical and horizontal axes stand for the UWES score and GPA category, respectively. The group was gender. The UWES scores still showed statistical significance in the women (*p* = 3.47e-38) and men (*p* = 2.36e-22) subgroups.

Moreover, to further eliminate the confounding effects of age and gender, we performed a subgroup analysis. The statistical significance of UWES still be demonstrated in subgroups of “age 16-20” and “age 21-25”, which further verified the positive correlation between UWES and GPA. Similarly, Figures 4C,D show that the UWES categories and scores remained statistically significant across gender subgroups. In addition, an overall trend of improved MSWE was observed as GPA increased. Consequently, the significant correlation between GPA and the UWES scores was further confirmed after eliminating the confounding effects of age and gender.

Above all, the results of the multivariate regression analysis and subgroup analysis provided insights by excluding the confounding effects of other variables in the nomogram, thereby focusing our research on GPA and further strengthening the conclusion. Accordingly, we confirmed the statistical significance of the correlation between GPA and the UWES scores.

### 3.4 Evaluative model for low UWES probability based on GPA

**Figure 5:**
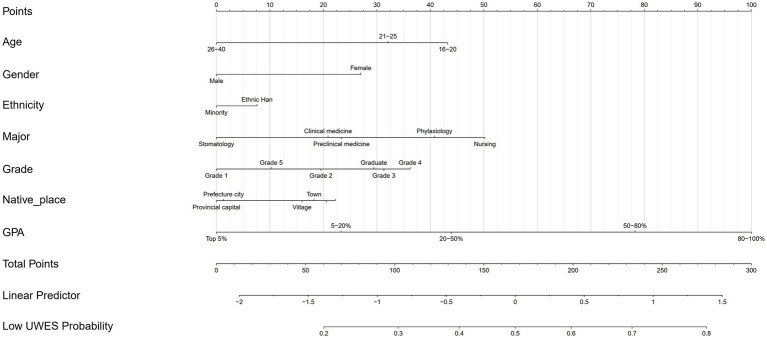
A nomogram was developed to evaluate the possibility of low UWES scores based on GPA and other key demographic variables. A higher GPA contributed to a lower point score, indicating a lower likelihood of having low UWES scores.

The nomogram (Figure 5) was constructed based on GPA coupled with demographic information (age, gender, ethnicity, and native place) and key medical education-related variables (major and grade) to evaluate the probability of low UWES scores, also known as worse MSWE.

Each variable contributed a different proportion when the total points (0–300 points) were calculated. The age variable accounted for 0 to 43 points, while gender contributed either 0 or 37 points to the total. Notably, it was surprisingly found that GPA accounted for up to 100 points, one-third of the maximum total, which meant that GPA was the predominant factor and had the strongest association with MSWE among all variables. However, a higher point corresponded to a greater likelihood of low UWES scores. Specifically, nursing (50 points) and grade 4 students (37 points) were more likely to have worse MSWE compared to other majors and grade levels, respectively. Notably, GPA’s contribution to the total points corresponded to the rank order sequentially. A worse GPA was projected to correspond with a higher point and a higher probability of low UWES scores, thereby leading to worse MSWE.

**Figure 6:**
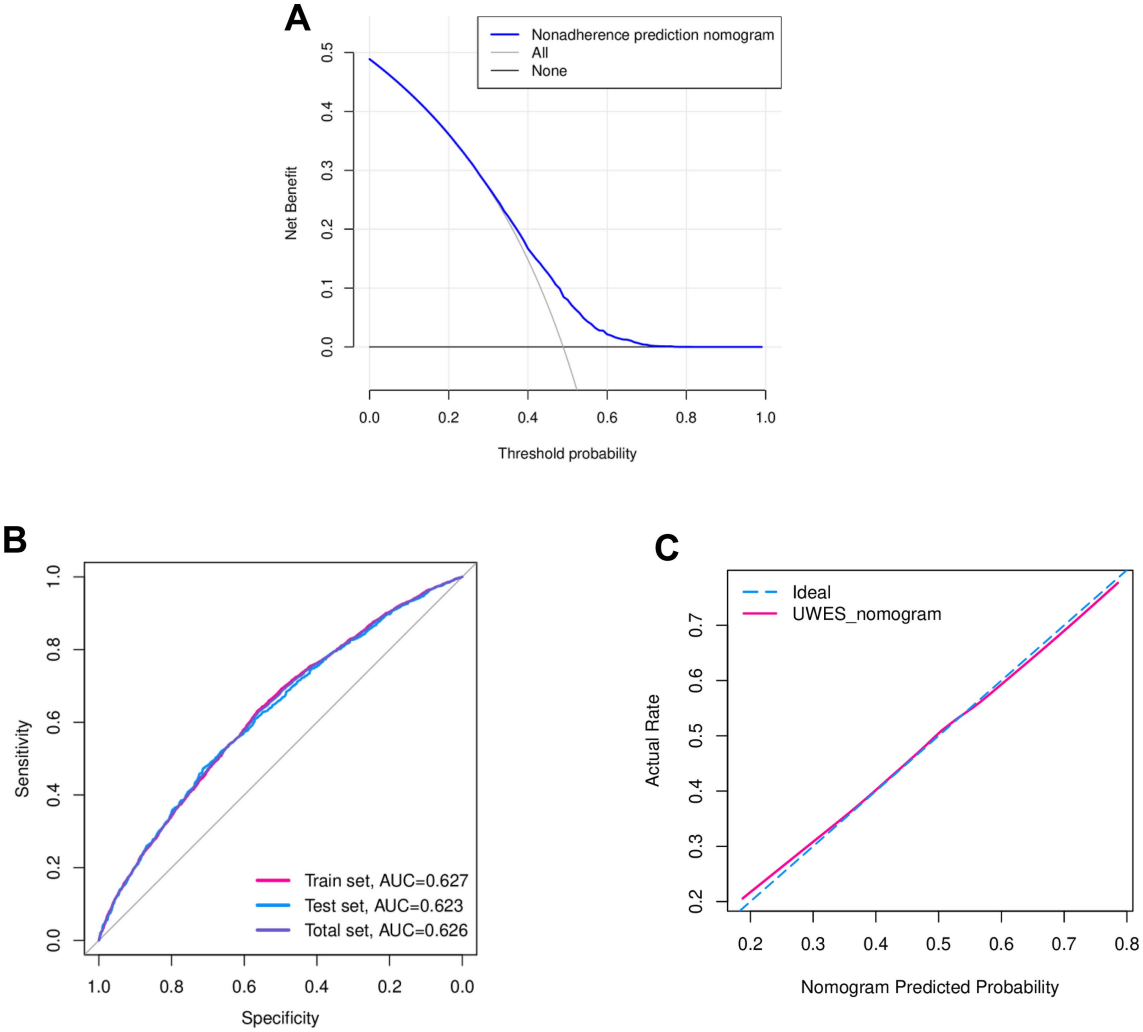
Validation of the nomogram. **(A)** DCA of the nomogram. Vertical and horizontal axes represent net benefit and threshold probability, respectively. The medical students had higher net benefits, especially when the low UWES probability exceeded 0.3. **(B)** ROC curve of the nomogram. Vertical and horizontal axes represent sensitivity and specificity, respectively. The ROC curve suggested potential evaluative discrimination and expected accuracy (Full set AUC = 0.626, training set AUC = 0.627, and test set AUC = 0.623). **(C)** The calibration curve of the nomogram. Vertical and horizontal axes represent the actual rate and the nomogram-predicted probability, respectively. The calibration curve showed a high degree of overlap between the ideal and UWES evaluative nomogram curves, indicating good agreement with the actual situation. DCA, decision curve analysis; UWES, Utrecht Work Engagement Scale; ROC, receiver operating characteristic; AUC, area under the curve.

The efficacy of this nomogram was checked using bootstrap internal validation, which was visualized through ROC and calibration curves. For DCA, the model’s curve is considered clinically useful if it lies above the reference curve across a range of relevant thresholds, indicating it reduces unnecessary interventions while identifying important cases. In Figure 6A, when the probability of low UWES exceeded 0.3, the non-adherence evaluation nomogram demonstrated a higher net benefit compared to all other models, highlighting the range and effectiveness of our model. In addition, for the ROC curve, the curve closer to the upper-left corner signifies stronger discriminative power. The area under the curve (AUC) quantifies overall performance, with 1 indicating perfect discrimination and 0.5 representing random chance. As shown in Figure 6B, the AUC of the ROC curves was 0.627, 0.623, and 0.626 for the training set, test set, and overall set, respectively, which suggested a relatively evaluative accuracy. Moreover, for the calibration curve, the evaluated probabilities are grouped into intervals. For each interval, the mean evaluated probability and the observed event rate were computed. Figure 6C shows a high degree of overlap between the ideal and UWES evaluative nomogram curves via the calibration curve, highlighting good agreement with the actual situation.

## 4 Discussion and conclusion

Medical students’ work engagement (MSWE) refers to a positive, fulfilling, and affective-motivational state of mind experienced by medical students during their learning and practice in school or hospital (8). In addition, medical students’ work performance and productivity can be reflected by MSWE (5, 6), which is specified as vigor, absorption, and dedication. For medical students, favorable MSWE also gives rise to positive reactions toward academic learning and research (28, 29). For the educational system, it is essential to implement effective higher education by improving MSWE (28). Therefore, it is critical to evaluate MSWE during learning and identify its factors.

The Utrecht Work Engagement Scale (UWES) was utilized to evaluate MSWE quantitatively. Our previous research systematically showed that GPA had a significant correlation with SRL (15), which combines the UWES dimensions of dedication and absorption. To explore the impact of medical students’ GPA on MSWE, we conducted a cross-sectional study where GPA and the UWES scores or categories were recorded at the same time.

The Pearson’s chi-squared test and Welch’s ANOVA revealed a statistically significant association between GPA and UWES scores. The results indicated that medical students with higher GPAs tend to demonstrate greater work engagement in learning and practice. Furthermore, GPA was demonstrated to be a significant factor influencing MSWE through multivariate logistic regression analysis, after controlling for the confounding effects of gender and age. Finally, a nomogram was constructed to evaluate the possibility of low UWES scores based on GPA and key demographic information.

**Figure 7:**
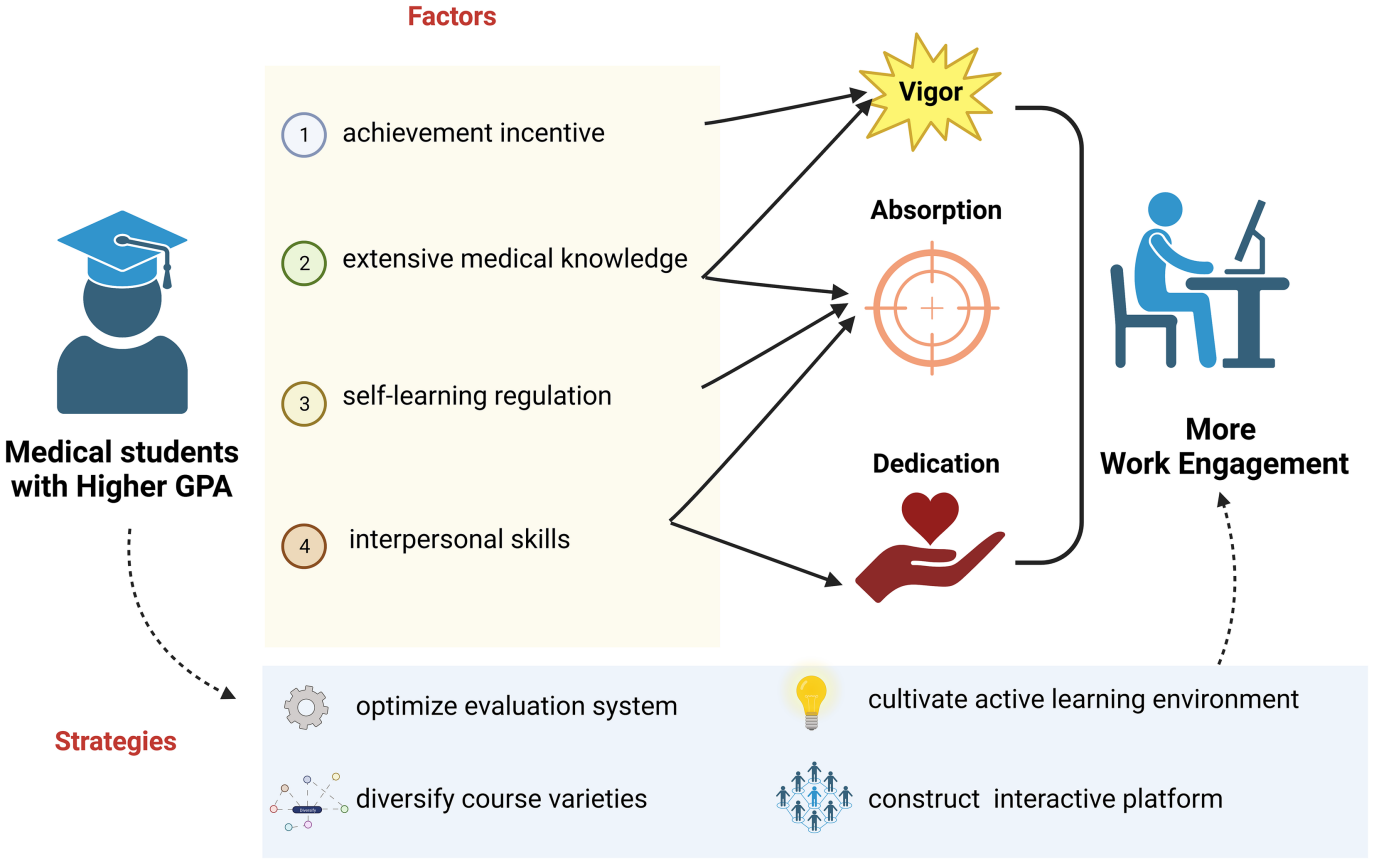
Collection and visualization of the discussion outlining the pathways through which GPA influences MSWE, along with relevant suggestions. The medical students with higher GPAs tended to possess achievement motivation, extensive medical knowledge, strong self-regulation skills, and interpersonal abilities, all of which enhance students’ vigor, absorption, and dedication—key components of MSWE. Educational instruments are strongly recommended to optimize the evaluation system, diversify course offerings, cultivate an active learning environment, and construct an interactive platform to improve MSWE. Created in BioRender. Zhou, J. (2025) https://BioRender.com/m4vut6v, licensed under Academic License.

Having established the statistically significant relationship between medical students’ GPA and MSWE, we will further discuss the reasons why medical students with higher GPAs are able to show greater work engagement in learning and practice (Figure 7). It is crucial to clarify the concepts of GPA and MSWE before the discussion. GPA is defined as an overall evaluation of a student’s academic performance, combining multiple curriculum assessments according to a specific weighting scheme (30). GPA can also reflect capabilities such as achievement motivation, self-regulation, extensive knowledge, and interpersonal communication. The verified validity of GPA is what makes it a widely used criterion among most universities (31). In addition, work engagement can be specified into three dimensions: vigor (emotional motivation), absorption (immersion in work), and dedication (making contributions) (26). Accordingly, we will clarify the interaction between the comprehensive capabilities reflected by GPA and medical students’ content of WE.

Initially, medical students with high GPAs often show achievement motivation in their professional field, which can be explained by the incentive derived from their past accomplishments (32). Importantly, this reflects a type of intrinsic motivation, especially when students are confronted with optimally challenging tasks. According to the theory of self-determination (33), intrinsic motivation enhances students’ vigor correspondingly once they are engaged in the work. Furthermore, students with achievement motivation are motivated by a sense of personal competence, which fosters a sense of responsibility and encourages greater engagement in work (34).

Furthermore, medical students with high GPAs are often considered to possess an extensive range of medical knowledge and clinical skills, as required by many medical schools. Such students, equipped with explicit knowledge, tend to show improvements in reaction time and execution quality (35), which can enhance their vigor for staying ahead and enthusiasm for tackling more challenges in their work. It is well known that medical students can achieve better learning outcomes via the gradual reinforcement of knowledge visualization (36). Equipped with extensive knowledge and clinical skills, medical students with high GPAs tend to concentrate on their work, rather than be frustrated by relatively burdensome tasks. Accordingly, they demonstrate a high level of absorption in learning, especially when confronted with challenges.

Meanwhile, medical students with higher GPAs are characterized by stronger self-regulation, excelling in setting appropriate goals and deploying effective strategies by drawing on a broad arsenal of metacognitive skills (15, 37). It is noteworthy that WE is not evaluated solely by the time dedicated. Instead, individuals’ efficacy and concentration during work also play a key role in evaluating MSWE. Although self-regulation enables medical students to respond quickly to changing clinical situations and adjust their treatment strategies accordingly (38), it also plays a positive role in enhancing individuals’ efficacy and concentration. Therefore, a high level of WE results from excellent medical students’ self-regulation in the form of efficient absorption.

In addition, high GPA often reflects outstanding interpersonal skills and a spirit of collaboration (39), since the evaluation of teamwork contributes to the grades of certain college courses, such as problem-based learning or case discussions in medical education (40). Furthermore, medical students with high GPAs usually unify the team through interpersonal communications, prioritizing the team’s interest above individual goals and demonstrating greater attentiveness in teamwork. Notably, team members are mutually influenced by each other. An overall atmosphere of dedication and absorption is thereby infused, which also has a counter-effect on the WE of high-GPA students.

Above all, there are numerous pathways that illustrate how GPA affects an individual’s WE. The key point is to propose practical strategies to improve MSWE. First, educational instruments are expected to optimize the evaluation system for each single course and diversify course offerings, which makes GPA a more convincing index of a student’s comprehensive capabilities. In this way, the effects of GPA on MSWE will be more explicit. Next, extrinsic motivation plays an important role in enhancing WE (41), which can be achieved by the cultivation of an active learning environment (42). Several student-centered learning modules should be advocated, including inquiry-based learning, team-based learning, case-based learning, and problem-based learning (43). Meanwhile, medical students are encouraged to engage in critical thinking and heated discussion. If needed, e-learning programs or software can also provide an interactive platform to achieve active learning (44), thus enhancing MSWE.

However, there are some limitations in our research. From the perspective of methodological limitations, due to the cross-sectional design, the study could not establish causality between GPA and work engagement. Longitudinal research is needed to examine how these variables interact over time. Furthermore, for the nomogram, its discriminative ability and accuracy are modest (AUC = 0.626), indicating that GPA alone cannot reliably predict MSWE. In fact, our primary aim was to explore associations between GPA and medical students’ work engagement, rather than to develop a robust predictive tool. The nomogram we developed served as a visualization tool for our multivariate logistic regression model, rather than a powerful predictive instrument. Anyway, it implies that we should optimize the model in the future to achieve higher accuracy in evaluation. Moreover, both GPA rankings and UWES responses were self-reported, which may introduce bias. The students may have overreported their work engagement due to perceived expectations in the questionnaire to get a higher UWES score. In addition, self-assessed academic rankings may not accurately reflect official records, which might have introduced recall bias. From a practical and cultural perspective, the nomogram is limited to serving as a preliminary screening tool to distinguish extremely low or high probabilities of MSWE, and it requires further external validation to consolidate its validity. Meanwhile, potential sampling bias should be noted. The first batch of Chinese medical schools accounted for more than half of the universities selected, which could hinder the generalizability of the findings. Within the context of the Chinese educational system, the applicability of these findings to most Western countries is limited. The strong emphasis on exam performance in China may amplify GPA’s role in medical students’ work engagement, compared to Western educational systems. Furthermore, the pressures associated with the “Double First-Class” policy may differentially affect engagement variability among mid-tier students. In addition, collectivist norms may bias the UWES responses toward dedication items while underreporting disengagement. These contextual factors suggest that GPA-based interventions may hold particular relevance in the Chinese context but would require careful cultural adaptation for application in other settings. Therefore, in the future, our team will conduct external validation across diverse medical schools to assess generalizability and incorporate objective GPA data and additional psychosocial variables to enhance evaluative power.

In conclusion, our study clarified the correlation between GPA and work engagement among medical students through a series of verifications. Although the mechanism of GPA toward MSWE requires further research and consolidation, our findings provide new insights into the reformation of medical education by enhancing MSWE.

## Data Availability

The original contributions presented in the study are included in the article/Supplementary material, further inquiries can be directed to the corresponding authors.

## Footnotes

^1^ https://www.wjx.cn/
