# Supplementary material for: Exploring the association between grade point average and work engagement: insights from a cross-sectional study of medical students
Source: Front Med (Lausanne). 2025 Oct 8;12:1536482. doi: 10.3389/fmed.2025.1536482 (PMC12542906; doi:10.3389/fmed.2025.1536482)
Supplement: Supplementary file 1 [file Table_1.docx]

**TABLE S1**丨Detailed items in UWES

| **Items** | **Answers** | | | | | | |
| --- | --- | --- | --- | --- | --- | --- | --- |
|  | **Never** | **Hardly** | **Seldom** | **Sometimes** | **Often** | **Usually** | **Always** |
| **Vigor** |  |  |  |  |  |  |  |
| 1. At my study, I feel bursting with energy. |  |  |  |  |  |  |  |
| 2. At my study, I feel strong and vigorous. |  |  |  |  |  |  |  |
| 3. When I get up in the morning, I feel like going to study. |  |  |  |  |  |  |  |
| 4. I can continue studying for very long periods at a time. |  |  |  |  |  |  |  |
| 5. At my study I always persevere, even when things do not go well. |  |  |  |  |  |  |  |
| **Dedication** |  |  |  |  |  |  |  |
| 6. I find the study that I do full of meaning and purpose. |  |  |  |  |  |  |  |
| 7. I am enthusiastic about my study. |  |  |  |  |  |  |  |
| 8. My study inspires me. |  |  |  |  |  |  |  |
| 9. I am proud on my specialty. |  |  |  |  |  |  |  |
| 10. To me, my study is challenging. |  |  |  |  |  |  |  |
| **Absorption** |  |  |  |  |  |  |  |
| 11. Time flies when I’m studying. |  |  |  |  |  |  |  |
| 12. When I am studying, I forget everything else around me. |  |  |  |  |  |  |  |
| 13. I feel happy when I’m studying intensely. |  |  |  |  |  |  |  |
| 14. I’m immersed in my study. |  |  |  |  |  |  |  |
| 15. I get carried away when I’m studying. |  |  |  |  |  |  |  |
| 16. It’s difficult to detach myself from my job. |  |  |  |  |  |  |  |
